# Deficits in color detection in patients with Alzheimer disease

**DOI:** 10.1371/journal.pone.0262226

**Published:** 2022-01-04

**Authors:** Hee Jin Kim, Jae Hyun Ryou, Kang Ta Choi, Sun Mi Kim, Jee Taek Kim, Doug Hyun Han

**Affiliations:** 1 Department of Psychiatry, College of Medicine, Chung-Ang University, Seoul, Republic of Korea; 2 Department of Ophthalmology, College of Medicine, Chung-Ang University, Seoul, Republic of Korea; Niigata University, JAPAN

## Abstract

Deficits in color vision and related retinal changes hold promise as early screening biomarkers in patients with Alzheimer’s disease. This study aimed to determine a cut-off score that can screen for Alzheimer’s dementia using a novel color vision threshold test named the red, green, and blue (RGB) modified color vision plate test (RGB-vision plate). We developed the RGB-vision plate consisting of 30 plates in which the red and green hues of Ishihara Plate No.22 were sequentially adjusted. A total of 108 older people participated in the mini-mental state examination (MMSE), Ishihara plate, and RGB-vision plate. For the analyses, the participants were divided into two groups: Alzheimer’s dementia (n = 42) and healthy controls (n = 38). K-means cluster analysis and ROC curve analysis were performed to identify the most appropriate cut-off score. As a result, the cut-off screening score for Alzheimer’s dementia on the RGB-vision plate was set at 25, with an area under the curve of 0.773 (*p*<0.001). Moreover, there was a negative correlation between the RGB-vision plate thresholds and MMSE scores (r = -0.36, *p* = 0.02). In conclusion, patients with Alzheimer’s dementia had a deficit in color vision. The RGB-vision plate is a potential early biomarker that may adequately detect Alzheimer’s dementia.

## Introduction

### Degenerative process as the leading cause of dementia

Dementia is defined as a disease characterized by progressive impairment of cognition and a consequential decline in social and occupational functioning [1]. There are several types of dementia based on etiology, such as degenerative dementia (e.g., Alzheimer’s disease, frontotemporal dementia, Parkinson’s disease, and Lewy body dementia), vascular dementia, and dementia caused by other medical and neurologic conditions or various substances [2]. Alzheimer’s disease (AD) is the most common cause of dementia, accounting for 60%–80% of all causes of dementia [3]. AD is characterized by degenerative changes such as brain atrophy, neuronal loss, and major loss of synapses due to the deposition of neurofibrillary tangles and amyloid-β plaques in the brain [4].

Early detection of dementia provides a number of advantages, including the prevention of risk factors and timely appropriate treatments [5, 6]. Although AD is currently incurable, there are pharmacologic and cognitive treatment options that alleviate dementia symptoms and slow disease progression [7]. Moreover, patients can establish their treatment plans for comorbid diseases in advance. They can also proactively cope with economic or legal issues prior to losing their judgment due to dementia [6, 8]. In addition, given the increasing global disease burden of dementia, early detection of dementia is essential for secondary prevention [9].

### Biological markers of AD

The final diagnosis of AD requires a neuropathological examination of the brain [10]. It is very difficult to examine neuropathology in a living patient in a clinical setting. However, a biomarker that could objectively reflect such brain pathology would be beneficial for clinical diagnosis and the evaluation of therapeutic interventions [10]. Currently, neuroimaging biomarkers (e.g., medial temporal lobe atrophy, decreased glucose metabolism in the temporo-parietal lobe, amyloid-β deposition) and neurochemical biomarkers (e.g., abnormal amyloid-β and tau proteins in cerebrospinal fluid and plasma) are widely used for the detection of AD [11–14]. In addition, the *ε*4 allele of the apolipoprotein E gene is a well-known genetic biomarker of AD [15].

However, to date, there are no established peripheral biomarkers that are simple, inexpensive, and readily available in clinical settings. Olfactory and retinal changes have been suggested as potential peripheral biomarkers of AD [7, 16]. Kim et al. developed a novel olfactory threshold test for dementia screening that adequately detected cognitive decline in older patients [7]. In addition, retinal changes are of great interest as potential biomarkers of AD [16].

### The degeneration of the retina and color vision impairment

The retina, which is derived from a part of the brain during neurodevelopment, consists of three layers of neurons: the outermost layer is composed of photoreceptor cells (rods and cones), and the second layer is composed of bipolar cells. These cells transmit the signals of photoreceptors to the third layer, the retinal ganglion cells (RGC) [17]. Cell bodies of RGCs comprise the retinal nerve fiber layer (RNFL), and axons of RGCs form the optic nerve. This is where synaptic connections via the dorsal lateral geniculate nucleus terminate in the visual cortex [18, 19].

Rods are responsible for scotopic vision and do not mediate color vision. Cone cells are responsible for photopic vision and are capable of color vision [20]. There are three different types of cones. Each responds differently to wavelengths of light, depending on the photopigment it contains: red, green, or blue [21]. Color vision deficiency (CVD) is a congenital or acquired condition in which one or more of these primary cone cells are absent or damaged [22]. Depending on the type of cone affected, the deficiencies are classified as red-green CVD or yellow-blue CVD [23]. The most common CVD is X-chromosome-linked red–green deficiency [24]. Accordingly, the most common form of the color vision test is the Ishihara plate, a type of pseudoisochromatic plate that detects red-green deficiency [25]. It is suggested that acquired red-green CVDs are caused by lesions in the RGC, optic nerve, visual pathway, and visual cortex. Acquired yellow-blue CVD results from lesions in the photoreceptors and outer plexiform layers [25].

In patients with AD, impairment of color vision due to retinal changes (e.g., RNFL thinning and morphological changes in the optic nerve) has been reported [26, 27]. The underlying mechanism of color vision impairment in AD patients has been suggested to result from a general loss of RGCs and the consequential alteration of the visual pathways responsible for color vision [16]. RGCs are particularly sensitive to neurodegenerative damage, such as defective mitochondrial dynamics, axonal transport, oxidative stress, and energy depletion. This is a result of their high metabolic demands [28]. Defects in the RNFL are now regarded as the earliest sign of AD that precedes hippocampal atrophy [29]. However, only a few studies have suggested that color vision impairment could be an early biomarker of AD [10, 16]. Chang et al. suggested that the Ishihara color vision test may be a potential ocular test for biomarkers of AD [16]. Arnaoutoglou et al. reported that red-green color vision impairment on the Ishihara test differentiated AD, a degenerative dementia, from vascular dementia (VD), a non-degenerative dementia [30]. However, previous studies screened AD in older participants and assessed the correction rate and time consumption in all 38 plates of the Ishihara test. Simple and effective tools that take less than 5 minutes to complete and require minimal attention are considered ideal dementia screening tools in clinical settings [16, 31]. Therefore, we aimed to develop a novel color vision threshold test for AD screening that is easily applicable in clinical settings. Based on previous studies, patients with AD are expected to present higher color vision thresholds, or in other words, lower sensitivity. Moreover, we aimed to determine a cut-off score that could identify individuals who are under the potential degenerative course of AD.

### Hypothesis

We hypothesized that patients with AD would present color vision impairment measured using the Ishihara plate test and with the newly developed red, green, and blue (RGB) modified color vision plate test (RGB-vision plate). In addition, participants’ color vision impairment levels would be correlated with neurocognitive impairments.

## Methods

### Participants

This cross-sectional study was conducted between October 2020 and May 2021 at the Department of Psychiatry of Chung-Ang University Hospital in Seoul, Korea. Patients with pre-existing Alzheimer dementia or vascular dementia, individuals on their first visit for cognitive assessment, and individuals who responded to a hospital bulletin advertisement were enrolled in the study. The inclusion criteria were (a) age > 60 years, (b) awareness of the study, and (c) having provided informed consent. The exclusion criteria were as follows: (a) any past or current neurological (e.g., brain tumor, epilepsy, and Parkinson’s disease) or psychiatric disease (e.g., major depressive disorder, bipolar disorder, or schizophrenia) other than neurocognitive disorder based on the Structured Clinical Interview for DSM-5 Disorders-Clinician Version (SCID-5-CV) [32]; (b) any past or current diagnosis of dementia other than Alzheimer’s dementia or vascular dementia (e.g., Lewy body dementia, frontotemporal dementia, Parkinson’s disease dementia,); (c) a history of stroke or head trauma; (d) color blindness, color amblyopia, acute optic neuritis, or a history of macular degeneration or glaucoma; (e) communication difficulties resulting from severe hearing impairment or aphasia; and (f) failure to understand the study protocol and objectives. In regards to the participants’ capacity to understand and provide consent to the study, we presented the participants with an informative handout with the research information, in which they were able to make additional inquiries if necessary. This study was approved by the Institutional Review Board of the Chung-Ang University Hospital (approval number: 1922-015-400). Written informed consent was obtained from each participant or caregiver (spouse or adult child).

Of the 132 initially screened individuals, three individuals who did not understand the study protocol and objectives, five who had communication difficulties, eight who had glaucoma, three who had a history of stroke or head trauma, and five who had neurologic or psychiatric disease (Parkinson’s disease, brain tumor, schizophrenia, major depressive disorder) were excluded. Thus, 108 participants completed the study and were included in the final analysis.

### Clinical evaluation and procedures

#### Mini-Mental State Examination (MMSE)

A Korean version of the Mini-Mental State Examination (MMSE-K) [33] was used to evaluate cognitive impairment in participants. The MMSE-K provides quantified information on overall cognitive function within 5 to 10 minutes. The MMSE-K consists of the following items: registration (repeating prompts), attention and calculation, recall, language, ability to follow simple commands, and orientation [34]. The advantages of using the MMSE-K include the requirement of no specialized equipment or training for administration and having both validity and reliability for the diagnosis and longitudinal evaluation of AD. The limitation of the MMSE-K is that it is influenced by demographic factors, particularly age and education backgrounds.

#### Ishihara plate test

A paper version of the Ishihara 38 plate test was provided to every participant to evaluate color vision impairment and its severity. The Ishihara test detects color vision defects in the protan (red) and the deutan (green) axes, but not in the tritan axis (blue) [35]. The Ishihara test consists of 38 pseudoisochromatic plates, each with a circle of colored dots and a number or line inside the circle [36]. There are many types of demonstration plates designed to be visible to all people. These include transformation plates that are seen differently by people with color vision impairment, vanishing plates invisible to people with color vision impairment, hidden digit plates visible only to people with color vision impairment, diagnostic plates to determine the type of color vision deficit and its severity, and tracing plates where the participants are asked to trace a visible line across the plate instead of reading the numbers [36].

#### RGB modified color vision plate

**Fig 1:**
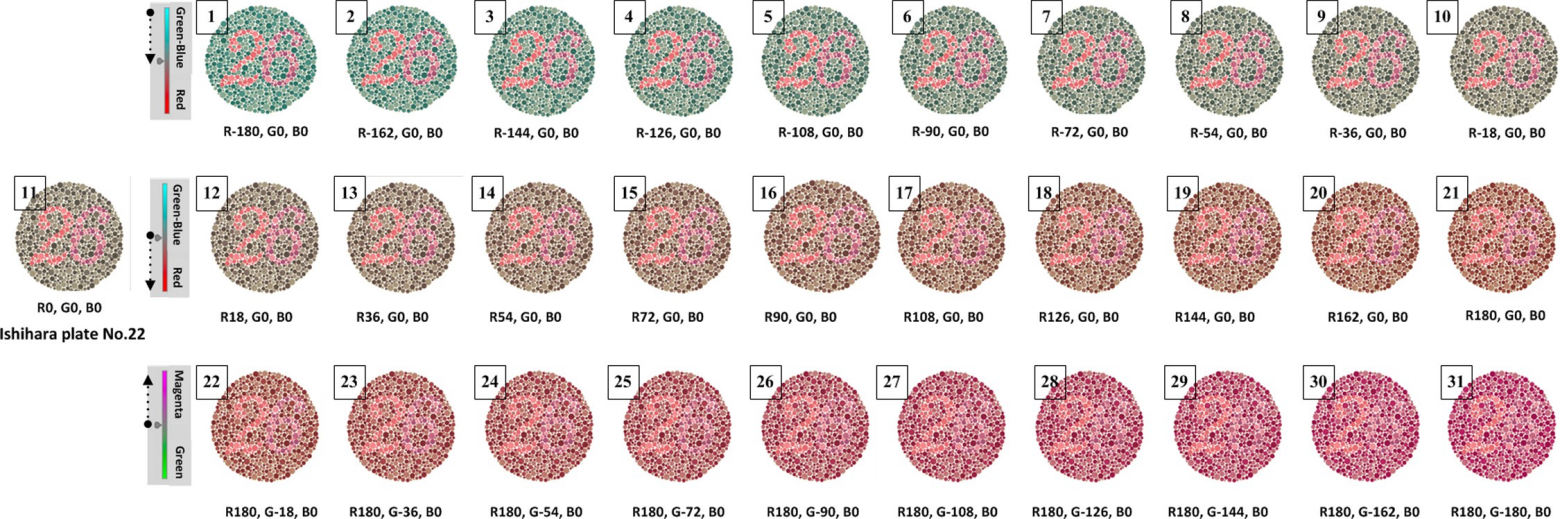
RGB-vision plate.

We developed a novel color vision threshold test called the RGB-vision plate. This test was performed by adjusting the hues of red and green colors of the Ishihara plate No. 22 using Adobe Photoshop CS6 software (Adobe Systems, San Jose, CA, USA). Ishihara plate No. 22 is a diagnostic plate that is to determine the type of color vision deficit and its severity [36]. The RGB-vision plate consists of 31 plates of a threshold series. The hues were adjusted sequentially first for red in 20 steps from -180 to +180 for red, and then for green in 10 steps from 0 to -180 with red fixed at +180. Each plate was numbered from plate No. 1 (red-180, green0, blue0) to plate No. 31 (red180, green-180, blue0) (Fig 1). We did not adjust hues for green from 0 to +180, as this range of hues overlapped with hues for red from -180 to 0.

RGB-vision plates were used to assess the color vision threshold of each participant. The participants were asked to read the hue-adjusted plates in reverse order, starting with plate No. 31, until the numbers of the plate were visible. Lower correction scores of RGB-vision plates may indicate more deficits in color vision.

### Statistical analysis

Demographic characteristics and MMSE scores were analyzed using one-way analysis of variance (ANOVA) and chi-squared tests. Between-group comparisons of Ishihara plate scores and RGB-vision plate scores were performed using one-way ANOVA. The correlations between MMSE scores and Ishihara total scores as well as the correlations between MMSE scores and RGB-vision plate scores were determined in each group (Alzheimer Dementia, Vascular Dementia, Healthy Control).

A K-means cluster analysis was performed to allocate the participants to either the Alzheimer dementia or healthy control group. Receiver operating characteristic (ROC) curves for each of the Ishihara plate scores and RGB-vision plate scores were used to calculate the sensitivity, specificity, and area under the ROC curve (AUC). They were also used to determine the cut-off value for identifying individuals with AD. The value with the highest sensitivity and specificity was determined as the best cut-off point. All statistical analyses were performed using SPSS ver. 19.0 (IBM Corp., Armonk, NY, USA).

## Results

### Demographic characteristics

There were no significant differences in age, sex ratio, education years, or prevalence of DM, hypertension, and hyperlipidemia between patients with AD, patients with vascular dementia and healthy comparison participants (Table 1). However, there was a significant difference in MMSE scores between the two groups. Patients with AD showed the lowest MMSE scores, compared to patients with vascular dementia and healthy controls (Table 1).

**Table 1:** Demographic characteristics.

	Alzheimer Dementia (n = 42)	Vascular Dementia (n = 28)	Healthy Control (n = 38)	Statistics
Age	78.1±5.8	77.6±5.6	76.2±4.5	F = 1.42, *p* = 0.26
Sex (M/F)	14/28	5/23	12/26	χ^2^ = 1.27, *p* = 0.26
Education (years)	7.0±6.0	7.8±6.1	8.9±4.3	F = 1.19, *p* = 0.31
DM (yes/no)	9/33	5/23	8/30	χ^2^ = 0.09, *p* = 0.79
HTN (yes/no)	25/17	14/14	21/17	χ^2^ = 0.13, *p* = 0.72
HyLPD (yes/no)	4/38	7/21	3/35	χ^2^ = 3.79, *p* = 0.06
MMSE scores ^ⱡ^	17.4±6.3	21.9±4.6	25.8±3.4	F = 28.0, *p*<0.01

ⱡ: post hoc test: Healthy Control > Vascular dementia > Alzheimer Dementia

DM, history of diabetes mellitus; HTN, history of hypertension; HyLPD, history of Hyper-lipidemia; MMSE, mini-mental state examination

### The comparison of Ishihara plate scores and RGB-vision plate scores between Alzheimer dementia, vascular dementia, and healthy controls

**Fig 2:**
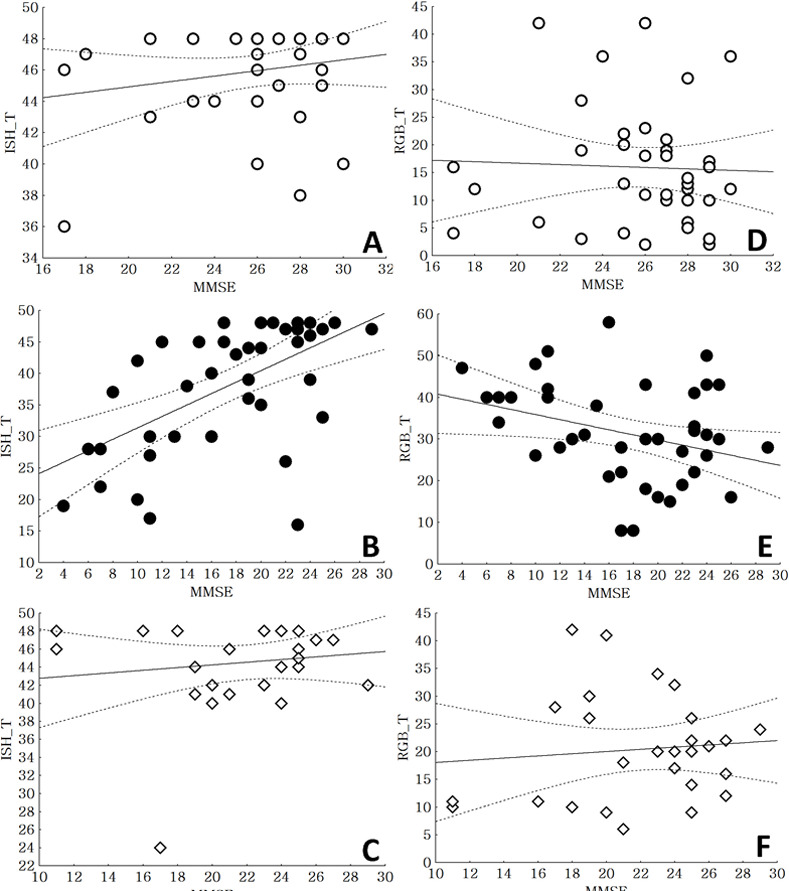
Correlations between MMSE scores and color detection scores. A: Correlation between mini-mental state examination (MMSE) scores and the Ishihara total score in healthy controls, r = 0.19, *p* = 0.25; B: Correlation between MMSE scores and the Ishihara total score in Alzheimer dementia, r = 0.58, *p*<0.01; C: Correlation between MMSE scores and the Ishihara total score in vascular dementia, r = 0.14, *p* = 0.46; D: Correlation between MMSE scores and the RGB total score in healthy controls, r = -0.04, *p* = 0.81; E: Correlation between MMSE scores and the RGB total score in Alzheimer dementia, r = -0.36, *p* = 0.02; F: Correlation between MMSE scores and the RGB total score in vascular dementia, r = 0.09, *p* = 0.63.

Patients with AD showed decreased correction scores on the Ishihara plate for the left eye, right eye, and in total compared to patients with vascular dementia and healthy controls (Table 2). There were positive correlations between MMSE scores and correction scores on the Ishihara plate in total (r = 0.58, *p*<0.01) (Fig 2).

**Table 2:** The comparison of Ishihara plate scores and RGB-vision plate scores between Alzheimer Dementia, vascular dementia, and healthy controls.

	Alzheimer Dementia (n = 42)	Vascular Dementia (n = 28)	Healthy Controls (n = 38)	Statistics
Ishihara Total^ⱡ1^	38.1±9.9	44.5±4.9	45.9±3.1	F = 14.29, *p*<0.01
Ishihara left^ⱡ2^	19.2±5.3	22.3±2.6	23.0.9±1.6	F = 11.74, *p*<0.01
Ishihara right^ⱡ3^	18.9±5.8	22.3±2.5	22.9±1.7	F = 11.87, *p*<0.01
RGB total^ⱡ4^	31.4±11.8	20.4±9.5	15.9±10.8	F = 21.11, *p*<0.01
RGB left^ⱡ5^	15.0±6.6	10.6±5.5	8.6±5.9	F = 11.32, *p*<0.01
RGB right^ⱡ6^	16.4±6.2	9.5±4.9	7.4±5.8	F = 26.66, *p*<0.01

post hoc test: ^ⱡ1^Healthy Control, Vascular dementia > Alzheimer Dementia;

^ⱡ2^Healthy Control, Vascular dementia > Alzheimer Dementia;

^ⱡ3^Healthy Control, Vascular dementia > Alzheimer Dementia;

^ⱡ4^Healthy Control, Vascular dementia < Alzheimer Dementia’

^ⱡ5^Healthy Control, Vascular dementia < Alzheimer Dementia;

^ⱡ6^Healthy Control, Vascular dementia < Alzheimer Dementia

Patients with AD showed increased correction scores on the RGB-vision plate for the left eye, right eye, and in total compared to patients with vascular dementia and healthy controls (Table 2). There were negative correlations between MMSE scores and correction scores on the RGB-vision plate (r = -0.36, *p* = 0.02) (Fig 2).

### Cluster analysis and characteristic curve analysis of the Ishihara plate scores and RGB-vision plate scores

In the K-means cluster analysis of the 80 participants (patients with AD and healthy controls), the highest (48) and lowest (16) Ishihara plate scores were selected as initial seeds (centroids of respective groups). The final centroid and standard deviation (mean ± SD) of the Ishihara plate scores of the patients with AD and healthy controls were 25.79 ± 6.00 and 45.23 ± 3.40, respectively. The between-group final Euclidean distance was 19.44. After K-means cluster analysis, we allocated 80 participants to patients with AD (14) or healthy controls (66).

**Fig 3:**
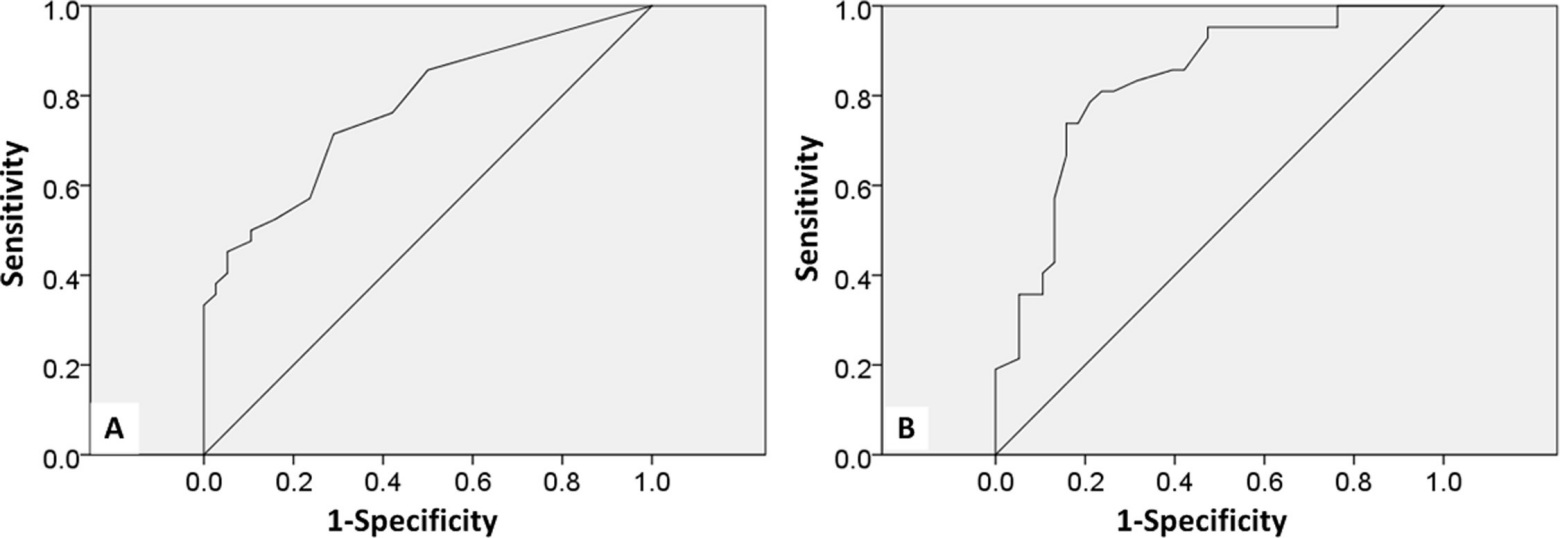
Receiver operating curve of Ishihara plate and RGB-vision plate. A: Ishihara plate; B: RGB-vision plate.

Among the 80 participants (patients with Alzheimer’s disease and healthy controls), the cut-off Ishihara plate score in patients with AD was 45, with an AUC of 0.773 (95% CI: 0.672–0.874, *p* < 0.001) (Fig 3). As shown in Table 3, the sensitivity and specificity values were highest for the Ishihara plate score of 45.

**Table 3:** Sensitivity and specificity of the Ishihara plate scores.

Cut-off value	True positive	False positive	False negative	True negative	Positive predictive value	Sensitivity	Accuracy	Negative predictive value	Specificity	Precision	Sensitivity + specificity
44	24	9	18	29	0.727	0.571	0.663	0.617	0.763	0.727	1.335
**45**	**30**	**11**	**12**	**27**	**0.732**	**0.714**	**0.713**	**0.692**	**0.711**	**0.732**	**1.425**
46	32	16	10	22	0.667	0.762	0.675	0.688	0.579	0.667	1.341

In the K-means cluster analysis of the 80 participants (patients with AD and healthy controls), the highest (58) and lowest (2) RGB-vision plate scores were selected as initial seeds (centroids of respective groups). The final centroid and standard deviation (mean ± SD) of the RGB-vision plate scores of the patients with AD and healthy controls were 36.54 ± 8.07 and 13.28 ± 6.26, respectively. The between-group final Euclidean distance was 23.26. After K-means cluster analysis, we allocated 80 participants to patients with AD (37) or healthy controls (43).

Among the 80 participants (patients with AD and healthy controls), the cut-off RGB-vision plate scores in the patients with AD was 25, with an AUC of 0.831 (95% CI: 0.739–0.922, *p* < 0.001) (Fig 3). As shown in Table 4, the sensitivity and specificity values were the highest for the RGB-vision plate scores of 25.

**Table 4:** Sensitivity and specificity of the RGB-vision plate scores.

Cut-off value	True positive	False positive	False negative	True negative	Positive predictive value	Sensitivity	Accuracy	Negative predictive value	Specificity	Precision	Sensitivity + specificity
23	31	7	11	31	0.816	0.738	0.775	0.738	0.816	0.816	1.554
**25**	**31**	**6**	**11**	**32**	**0.838**	**0.738**	**0.788**	**0.744**	**0.842**	**0.838**	**1.580**
27	29	6	13	32	0.829	0.690	0.763	0.711	0.842	0.829	1.533

## Discussion

The current study showed that a novel color vision test, termed the RGB-vision plate, reliably differentiated AD from healthy older individuals. The cut-off score of the RGB-vision plate for AD was set at 25. An increased red-green color vision threshold with a score of 25 or higher in the RGB-vision plate may be useful as an AD indicator. Although the conventional Ishihara plate test also differentiated AD and healthy controls with good sensitivity (0.714) and specificity (0.711), the RGB-vision plate showed better sensitivity (0.738) and specificity (0.842). Furthermore, considering the simplicity of the RGB-vision plate compared to other conventional color vision tests, the RGB-vision plate could be a useful early screening tool for detecting AD in older individuals.

In a correlation between MMSE and RGB-vision plate scores, AD patients with decreased MMSE scores showed a significantly increased color vision threshold, or decreased red-green color vision. This indicates that RGB-vision plate scores represent the progression of AD. These findings are consistent with previous studies on color vision impairment in patients with AD [16, 27, 30]. Color vision impairment in AD patients is considered to occur with global loss of RGC and thinning of the RNFL following the degenerative process [16]. This is further supported by studies that found amyloid-β plaques and neurofibrillary tangles in patients with AD. This may contribute to RGC death and the consequential thinning of the RNFL as well as morphological changes in the optic nerve [26, 37, 38]. Recent studies have shown altered amyloid precursor protein (APP) processing and fibrillary amyloid-β deposition in all six layers of the neuroretina and retinal vasculature [39, 40]. It has also been shown that retinal plaques preceded plaque deposition in the brain, which suggests that the amyloid-β deposition seen among ganglion cells could be an early sign of AD development [40]. In addition, hyperphosphorylated tau has been shown to accumulate in the RNFL [41, 42]. Thus, these findings indicate that abnormally processed and aggregated proteins such as APP or tau may be play a key role in causing the pathology visible in the eyes of AD patients [16]. Moreover, this explanation is in line with Köllner’s rule. This rule states that changes in the RGC, optic nerve, visual pathway, and visual cortex result in red-green deficiencies, whereas alterations of the outer retina and media contribute to yellow-blue CVD [25].

The clinical implication of this study is that a novel color vision threshold test was developed by adjusting the hues of the most used red-green color vision test. The cut-off value for screening AD was also determined using this test.

This study has several limitations. First, the RGB-vision plate could detect impairments in the protan and deutan axes, but not in the tritan axis. As some studies report tritan errors in AD patients [43], future investigations should include yellow-blue color screening tests such as the Hardy-Rand-Rittler test [44]. Second, this study did not directly investigate the association between color vision impairment and biological findings, such as retinal changes or cortical atrophy. Further studies, including biological assessments, such as optical coherence tomography (OCT) and magnetic resonance imaging (MRI), are underway to support the results of this study.

## Conclusions

The RGB-vision plate has been suggested as an effective tool for screening for AD. A high color vision threshold measured at an RGB-vision plate score of 25 or higher indicates possible AD. This tool can be used by physicians for early screening for AD in clinical settings. Further studies are required to determine the correlation between the RGB-vision plate and other diagnostic tools for AD to compare the diagnostic accuracy of the RGB-vision test with conventional screening tools.

## Supporting information

S1 Data (SAV)
